# Benchmark dataset of memes with text transcriptions for automatic detection of multi-modal misogynistic content

**DOI:** 10.1016/j.dib.2022.108526

**Published:** 2022-08-20

**Authors:** Francesca Gasparini, Giulia Rizzi, Aurora Saibene, Elisabetta Fersini

**Affiliations:** Department of Informatics, Systems and Communication, University of Milano-Bicocca, Italy

**Keywords:** Misogyny detection, Multi-modal content, Memes, Cybersexism, Visual and textual cues

## Abstract

In this paper we present a benchmark dataset generated as part of a project for automatic identification of misogyny within online content, which focuses in particular on memes. The benchmark here described is composed of 800 memes collected from the most popular social media platforms, such as Facebook, Twitter, Instagram and Reddit, and consulting websites dedicated to collection and creation of memes. To gather misogynistic memes, specific keywords that refer to misogynistic content have been considered as search criterion, considering different manifestations of hatred against women, such as body shaming, stereotyping, objectification and violence. In parallel, memes with no misogynist content have been manually downloaded from the same web sources. Among all the collected memes, three domain experts have selected a dataset of 800 memes equally balanced between misogynistic and non-misogynistic ones. This dataset has been validated through a crowdsourcing platform, involving 60 subjects for the labelling process, in order to collect three evaluations for each instance. Two further binary labels have been collected from both the experts and the crowdsourcing platform, for memes evaluated as misogynistic, concerning aggressiveness and irony. Finally for each meme, the text has been manually transcribed. The dataset provided is thus composed of the 800 memes, the labels given by the experts and those obtained by the crowdsourcing validation, and the transcribed texts. This data can be used to approach the problem of automatic detection of misogynistic content on the Web relying on both textual and visual cues, facing phenomenons that are growing every day such as cybersexism and technology-facilitated violence.

## Specification Table

**Table:** 

Subject	Artificial intelligence
Specific subject area	Automatic detection of multi-modal content
Type of data	Tables (.csv)
	Images (.jpeg)
How data were acquired	Images of memes were downloaded from the Web and saved in high quality JPG format with maximum dimension resized to 640 pixels in order to have a dataset of normalized dimensions; labelled data were collected from domain experts and through a crowdsourcing platform; text data were manually transcribed.
Data format	Raw data
	Analyzed data (labeled and transcribed)
Parameters for data collection	Memes were downloaded from popular social media platforms and websites dedicated to the collection and creation of memes, using keywords related to misogyny. The memes were subsequently manually selected. Each meme was evaluated by three domain experts and by three subjects through a crowdsourcing platform.
Description of data collection	The 800 memes of the dataset were selected from a bigger collection, by a pool of three experts of the domain in order to be balanced between misogynistic and non-misogynistic ones. The dataset has been validated by using the Figure Eight crowdsourcing platform (now called Appen, https://appen.com/), involving 60 subjects, and the text of each meme has been manually transcribed.
Data source location	Raw data as well as crowdsourcing labels were collected from the Web
	Data were processed at:
	Institution:University of Milano-Bicocca
	City: Milan
	Country: Italy
Data accessibility	In a public repository:
	Repository name: github
	Data identification name: Misogynistic-MEME
	Direct URL to data: https://github.com/MIND-Lab/MEME
	Instructions for accessing these data:
	Data are password protected. Password will be provided after fulfilling a copyright notice.
Related research article	E. Fersini, F. Gasparini, S. Corchs, Detecting sexist MEME on the Web: A study on textual and visual cues. In: 2019 8th International Conference on Affective Computing and Intelligent Interaction Workshops and Demos (ACIIW). IEEE, 2019. p. 226–231.

## Value of the Data

• The present dataset is constituted by multimodal contents in the form of memes, which are widely used in the Web to convey misogyny with both textual and visual media. In fact, having a multimodal set of data is mandatory to build efficient Machine Learning (ML) techniques that can intercept and counteract online discrimination, cybersexism and violence, which have been spreading more frequently with the increase of women’s roles in society. Having that most of these contents are shared online and especially on social media platforms, the memes constituting the presented dataset are all collected from these sources to guarantee a real-life scenario.
• Researchers from different fields may benefit from the use of the presented dataset. Researchers interested in natural language processing and computer vision can exploit the provided social media data to build ML models by considering both textual and visual contents. For example, they could design algorithms for discrimination detection.
  Moreover, companies that develop artificial intelligent strategies to control social media activities may benefit from the use of these data to provide bots that are able to automatically remove potentially offensive content. Finally, social science researchers can study the connotation of gender discrimination in the Web.
• This dataset presents a data structure that can be adopted for the collection of further data on the topic to develop more robust machine learning techniques. In fact the automatic detection of misogynistic content is particularly challenging especially considering that: (i) misogynistic and non misogynistic memes can share the same visual content but a different text, and (ii) misogyny can be expressed by text, image or by their combination.
  This dataset can also be adopted to increase the quality of the labelling task, as it can be used as a gold standard to check the reliability of annotators on crowdsourcing platforms. Moreover, it can provide a good starting point to collect other discriminatory and offensive contents like racism and cyberbullying.
• This dataset will pave the way not only to the development of machine learning techniques able to promptly intercept on offensive content against women the Web, but also to stimulate other researchers in devoting their attention to this phenomenon, increasing the awareness and sensitivity to misogyny as well as to other forms of discrimination.
• This dataset provides the labels from both domain experts and subjects recruited within the population. The comparison between the two distributions is also significant, not only with respect to misogyny by itself, but also with respect to the perception of aggressiveness and irony.
• This dataset of memes is intended to face the misogynist phenomenon. Even though a related dataset has been proposed by Facebook AI for the Hateful Meme (HM) Challenge [1], it mainly provides synthetic memes automatically generated as benign confounders of similar hateful memes. Instead, we collected memes from social media platforms to provide a real-life scenario. Moreover, while the HM dataset is generally devoted to general hateful and non-hateful memes, without focusing on any specific types and targets of hate, our dataset is centered on women as target.

## 1 Data Description

In the latest years, misogyny has found in the Web a new and powerful way of diffusion together with the new phenomenon of cybersexism, where women are often victim of offensive messages and, in the most serious cases, of abuse and threats. Online platform providers have introduced policies to prevent offensive content. However, due to the speed of dissemination of messages in social media, systems able to automatically filter offensive content are urgently needed [2], [3]. While new opportunities for females have been opened on the Web, systematic inequality and discrimination offline is replicated in online spaces in the form of offensive contents against them [4], [5].

Memes are especially popular communication means for social media users, being able to efficaciously convey funny and/or ironic jokes [6]. A meme is defined as an image composed of a pictorial information on which a text is superimposed a posteriori by a human [6]. Given the characterization of memes, they have been progressively used to convey hate [7] and, in this specific dataset, we are interested in memes circulating in the Web and targeting women with sexist and aggressive messages [8], [9].

The choice to investigate the intrinsic nature of the memes in terms of irony and aggressiveness has been inspired by Chateau et al. [10] and Taecharungroj et al. [11]. In particular, our goal is to understand if there is a link between irony and aggressiveness that could lead to an increase of the virality of specific types of memes.

In order to develop efficient machine learning techniques able to automatically detect multi-modal misogynistic messages online, we here present a dataset composed of:

• **800 memes**, saved as jpeg images, resized to have the greatest dimension equal to 640 pixels. These memes are saved with a progressive unique ID.
• A **table** saved as a.csv file, where all the data collected are reported, according to the following structure:
  – *memeID*: unique identifier associated to the meme;
  – *text*: transcription of the text reported in the meme;
  – *misogynisticDE*: Boolean attribute related to the presence of misogynistic content as reported by the Domain Experts (DE);
  – *aggressiveDE*: Boolean attribute; in case of a misogynist meme it represents the presence of aggressiveness, as reported by the DE;
  – *ironicDE*: Boolean attribute; in case of a misogynist meme it represents the presence of irony, as reported by the DE;
  – *misogynisticCS*: Boolean attribute related to the presence of misogynistic content, as reported by the annotators of the CrowdSourcing platform (CS);
  – *aggressiveCS*: Boolean attribute; in case of misogynist meme it represents the presence of aggressiveness, as reported by the CS;
  – *ironicCS*: Boolean attribute; in case of misogynist meme it represents the presence of irony, as reported by the CS.
  – *confidence_M_CS*: agreement on the misogynist attribute among the CS;
  – *confidence_A_CS*: agreement on the aggressiveness attribute among the CS;
  – *confidence_I_CS*: agreement on the misogynist irony among the CS;

We underline that the labels of the 3 experts have an agreement of 100% for all the 800 memes, as it was the criterion to select them among all the downloaded memes. For the memes labelled by the crowd, 554 of them have 100% of agreement, while the remaining 246 memes have an average confidence equal to 67%.

**Fig. 1:**
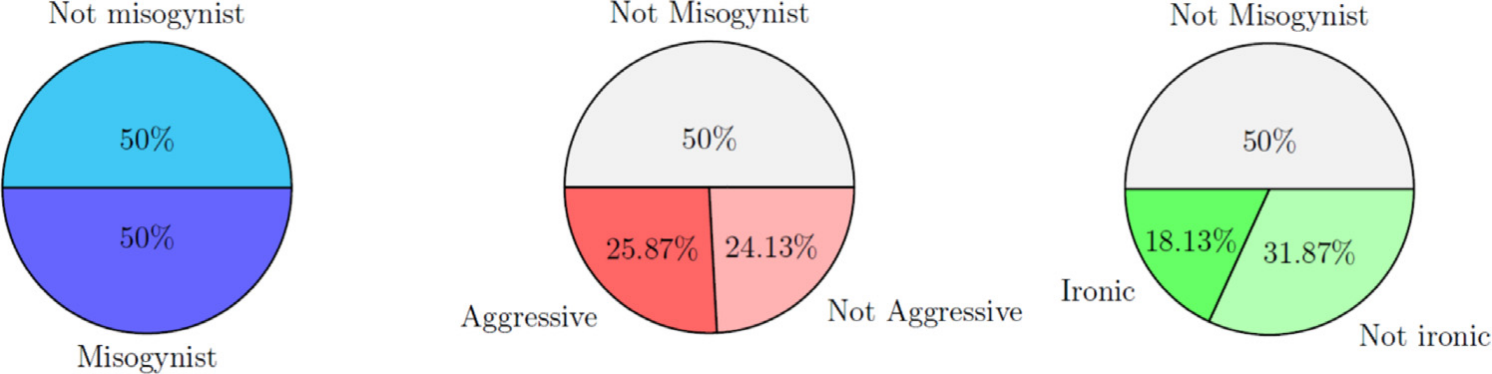
Distribution of the three labels given by the domain experts (DE).

*Data distribution*. The distribution of the three labels (misogyny, aggressiveness and irony) given by the domain experts are reported in Fig. 1. As the dataset of 800 memes was selected starting from the misogynistic DE labels, the first pie chart on the left confirms that the dataset is equally distributed within the two classes with 400 memes each. The second and third pie charts reported for the memes labelled as misogynistic the percentage of them considered respectively aggressive and ironic.

**Fig. 2:**
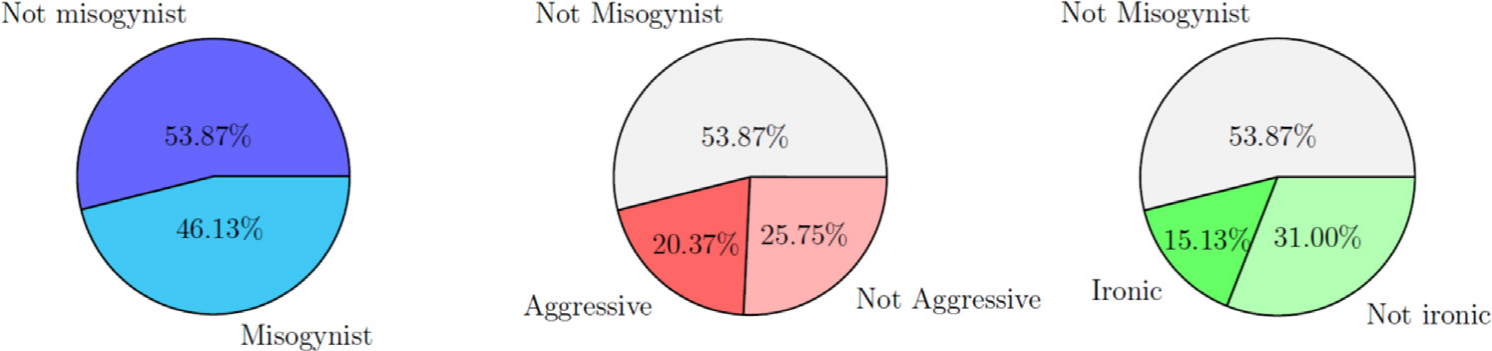
Distribution of the three labels given by the crowdsourcing annotators (CS).

In Fig. 2, the corresponding label distributions given by the CS annotators are reported. The first pie on the left shows that in this case the memes labelled as misogynistic are less than in the case of the DE evaluation.

Given the labels provided by DE and CS, 59 memes have been annotated differently with respect to the misogynistic label. Among them, 76.28% were considered misogynistic by the experts but not by the annotators, while only 23.72% of them were considered misogynistic only by the annotators. The complexity of the annotation process is also reflected by the agreements of the CS labellers, which have been reported in the corresponding columns in the Annotation Data sheet.

## 2 Experimental Design, Materials and Methods

### 2.1 Meme collection process

The most popular social media platforms, i.e. Facebook, Twitter, Instagram and Reddit, have been considered in the data collection phase. Memes that convey potential misogynistic content have been collected in the October–November 2018 period through the following operations [12]:

• Searching for threads or conversations dedicated and written by anti-women/feminists supporters, such as the Men Going Their Own Way (MGTOW) website and the related thread on Reddit;
• Exploring discussions on sexism in political or social events;
• Browsing hastags such as #girl, #girlfriend, #women.

Subsequently, the dataset has been enlarged by collecting memes from websites dedicated to meme creation and/or collection, as follows:

• Browsing hashtags such as #girl, #girlfriend, #women, #feminist;
• Consulting collections on all the variations of famous memes involving female characters.

In parallel, memes with non-misogynistic contents have been manually downloaded from the same web sources and by adopting the same keywords, for a non trivial collection of the memes.

### 2.2 Expert labelling and dataset definition

Three domain experts have evaluated all the collected memes, labelling them as misogynist or non-misogynist. The final dataset is composed of 800 memes, selected among those with an agreement of 100%, in order to have a perfect balanced dataset with respect to the two classes.

The experts have also annotated the misogynistic memes with respect to aggressiveness and irony. This phase provided the three corresponding boolean labels reported in the data sheet with DE suffix.

### 2.3 Dataset Annotation through the crowdsourcing platform

The 800 memes selected were labelled adopting a crowdsourcing platform (Figure Eight in 2018, now called Appen, https://appen.com/).

A controlled labelling experiment was chosen to provide judgments from trusted and reliable participants with equally distributed age (between 20 and 50 years old) and gender. All the data were anonymized.

The annotation task was designed as follows:

• the order of the memes in the experiment is randomized to avoid bias;
• the maximum number of judgments that any contributor can provide is limited to 40 memes, to limit fatigue and consequent unreliable annotations;
• any annotator can leave whenever he/she wants;
• for each annotator, the task expires after an hour and a half, regardless of the number of evaluated memes, in order to limit external stimuli;
• each annotation page shows only one meme at a time, to not influence or bias the participant by seeing other meme contemporaneously;
• each meme is evaluated by three different subjects.

**Fig. 3:**
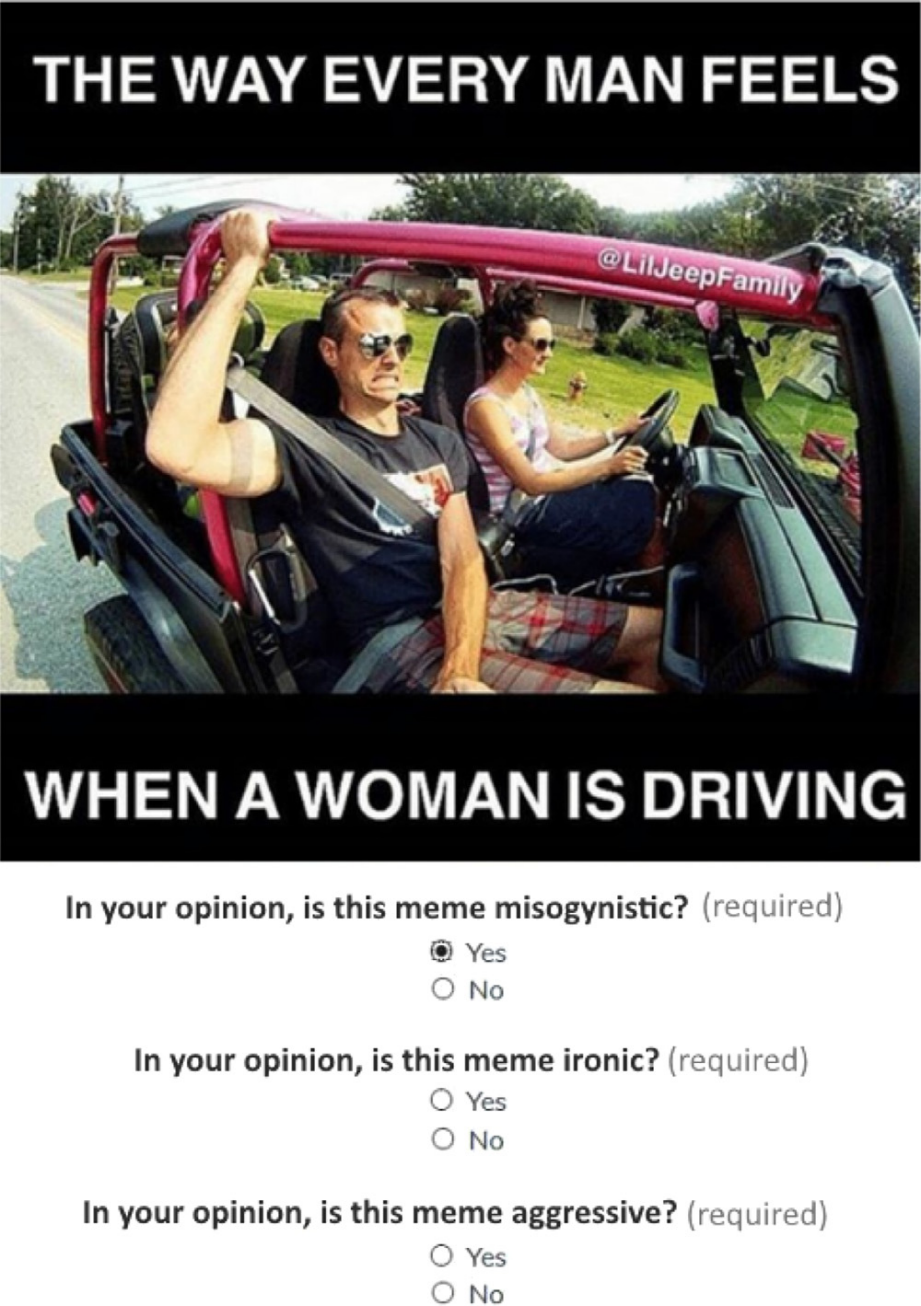
Questions asked in the crowdsourcing annotation task.

For each meme, questions were asked to the participants in English (Fig. 3). Firstly, the question **In your opinion, is this meme misogynistic?** is proposed to the participant.

Then, only in the case of a meme evaluated as misogynistic, the following two questions were proposed:

1. In your opinion, is this meme ironic?
2. In your opinion, is this meme aggressive?

The meme could be annotated as either, neither, or both ironic and/or aggressive.

Definitions and guidelines were not provided to the CS annotators, wanting to collect their perception of misogyny, irony and aggressiveness present in the meme, without influencing their decision. This crowdsourcing annotation provided the three corresponding boolean labels reported in the data sheet with CS suffix, together with the corresponding confidence level (in terms of percentage of agreement).

### 2.4 Text transcription

Finally, the text superimposed to the imaged have been manually transcribed for each of the 800 memes.

## Ethics Statement

The Ethics Committee of the University of Milano-Bicocca has approved the annotation task and the collection of the dataset (Protocol number 0064693). An informed consent was given to all the involved subjects, indicating the presence of possible explicit contents, explaining how to perform the tasks also reporting them the possibility to stop the labeling activity whenever they wanted. No personal data have been acquired to identify the labellers, being therefore GDPR compliant.

Moreover, the data gathering from social media platforms and other Websites does not intend to make any copyright infringement but is intended to be used for research purposes only. Therefore, the dataset requesters will be asked to fill an agreement to ensure the correct use of the presented dataset before being allowed to assess the data.

## CRediT authorship contribution statement

**Francesca Gasparini:** Conceptualization, Methodology, Investigation, Writing – original draft, Supervision. **Giulia Rizzi:** Software, Formal analysis, Data curation, Writing – original draft, Visualization. **Aurora Saibene:** Investigation, Data curation, Writing – original draft, Visualization. **Elisabetta Fersini:** Project administration, Conceptualization, Methodology, Investigation, Writing – original draft, Supervision.

## Declaration of Competing Interest

The authors declare that they have no known competing financial interests or personal relationships which have, or could be perceived to have, influenced the work reported in this article.

## Data Availability

Benchmark dataset of memes with text transcriptions for automatic detection of multi-modal misogynistic content (Original data) (Mendeley Data).
